# Specific nasal provocation test with dermatophagoides pteronyssinus monitored by acoustic rhinometry in children and adolescents with allergic rhinitis and controls

**DOI:** 10.1186/1939-4551-8-S1-A179

**Published:** 2015-04-08

**Authors:** Fausto Matsumoto, Gustavo Falbo Wandalsen, Dirceu Sole

**Affiliations:** 1 Department of Pediatrics, Division of Allergy, Rheumatolology and Clinical Immunology, Federal University of São Paulo, São Paulo/SP, Brazil; 2 Federal University of Sao Paulo, Brazil; 3 Brazilian Society, Brazil

## Background

Specific nasal provocation tests (NPTs) are indicated in confirming clinically relevant allergy and in the diagnosis of allergic rhinitis. Our objective was to evaluate a *Dermatophagoides pteronyssinus* (Dp) NPT protocol monitored by Acoustic Rhinometry (AR) and nasal symptom score in children and adolescents.

## Methods

Seventeen patients with allergic rhinitis sensitized to *Dermatophagoides pteronyssinus* and 15 controls were submitted to NPT with Dp. Acoustic Rhinometry was performed after bilateral instillation of 0.15ml of nasal saline and Dp, 5.000 UBE/ml (1:10000; 1:1000; 1:100; 1:10) until 20% fall in nasal volume in the segment between 0 and 5cm (V5) or nasal symptom score > 3 (0 to 11).

## Results

Median age was 122 months (108 to 143 months) in controls and 142 months (117 to 156 months) in allergic rhinitis group. At the end of the NPT, the mean V5 fall was 5.7% (-8.7% to 4%) in controls and 22.8% (-24% to -20%) in allergic rhinitis group. None of the 15 controls and 88% (15 of 17 patients) of the allergic rhinitis group had a positive Dp NPT. Considering positive NPTs, 23.5% (4/15) were positive at 1:10000, 35% (6/15) at 1:1000, 23.5% (4/15) at 1:100 and 6% (1/15) at 1:10 concentration. One NPT was considered positive due to symptom score. None of the 32 patients presented bronchial reactions or any pulmonary symptom after NPT.

## Conclusions

This protocol has showed to have good specificity and sensitivity to discriminate patients with allergic rhinitis from controls. A simplified protocol with two Dp concentrations (1:1000 and 1:100) seems to be less expensive and less time consuming NPT protocol to be applied in the clinical practice.

